# Editorial: Exploring miRNA roles in cancer pathogenesis and treatment, volume III

**DOI:** 10.3389/fmolb.2026.1833720

**Published:** 2026-04-17

**Authors:** Qinan Yin

**Affiliations:** Department of Radiation Oncology, Cancer Institute, The First Affiliated Hospital, and College of Clinical Medicine of Henan University of Science and Technology, Luoyang, China

**Keywords:** biomarkers, cancer pathogenesis, MicroRNAs, therapeutic targets, tumor microenvironment

MicroRNAs (miRNAs) are small non-coding RNAs that regulate gene expression at the post-transcriptional level and control key processes such as proliferation, apoptosis, and immune responses. Their dysregulation is a hallmark of cancer, where they function as oncogenes or tumor suppressors to shape tumor initiation, progression, metastasis, and therapy resistance. Owing to their stability in tissues and body fluids, miRNAs have also emerged as promising biomarkers and therapeutic targets (Calin and Croce, 2006).

Building on previous volumes, this third installment highlights recent advances in miRNA research in cancer. The four contributions in this Research Topic provide complementary perspectives spanning tumor-specific regulation, signaling networks, biomarker development, and RNA regulatory mechanisms.

A central theme emerging from this Research Topic is the role of miRNAs in tumorigenesis within specific cancer contexts. The review by Romani et al. summarizes current knowledge on miRNA involvement in adult ocular tumors, particularly uveal melanoma. The authors demonstrate how dysregulated miRNA networks contribute to tumor development, metastatic behavior, and clinical outcomes. Importantly, they highlight the potential of miRNAs as diagnostic and prognostic biomarkers detectable in ocular tissues and fluids, as well as their promise as therapeutic targets. The discussion of miRNA-based nanomedicine further illustrates how advances in delivery systems may facilitate the clinical translation of miRNA-targeted therapies.

Beyond tumor-specific settings, miRNAs function as central regulators of major oncogenic signaling pathways. Gupta et al. provide an integrated overview of the interplay between miRNAs, WNT signaling, and flavonoids. Their work emphasizes the bidirectional regulation between miRNAs and WNT pathway components, which shapes tumor growth, metastasis, and therapy resistance. Notably, the authors highlight flavonoids as modulators of miRNA expression, introducing a novel therapeutic perspective in which natural compounds can reprogram oncogenic signaling networks via miRNA regulation. This integrative framework supports the development of multi-targeted therapeutic strategies combining pathway modulation and epigenetic regulation.

The translational relevance of miRNAs is further exemplified in breast cancer. Baylie et al. review the role of miRNAs as diagnostic and prognostic biomarkers, as well as regulators of key oncogenic pathways such as PI3K/AKT signaling. By influencing proliferation, invasion, metastasis, and apoptosis, miRNAs contribute to multiple hallmarks of breast cancer. Importantly, circulating miRNAs-including miR-9, miR-10b, and miR-155-are highlighted as promising non-invasive biomarkers detectable in body fluids. These findings underscore the growing importance of miRNA-based liquid biopsy approaches in precision oncology.

Complementing these miRNA-focused studies, Kalathiya and Padariya provide mechanistic insights into RNA regulation through their investigation of the nonsense-mediated mRNA decay (NMD) pathway. Using molecular dynamics simulations, the authors reveal how cancer-associated mutations can alter protein–mRNA interactions and affect mRNA surveillance. Although not exclusively centered on miRNAs, this study expands the conceptual framework of RNA-based regulation in cancer, highlighting the interplay between mRNA quality control systems and post-transcriptional regulatory mechanisms.

Collectively, these contributions highlight miRNAs as central regulators linking signaling pathways, epigenetic control, and tumor behavior. Advances in high-throughput sequencing and bioinformatics continue to accelerate the identification and functional characterization of miRNAs, while their utility as minimally invasive biomarkers and therapeutic targets is rapidly expanding (Rupaimoole and Slack, 2017).

Despite this progress, several challenges remain, including limited delivery efficiency, potential off-target effects, and the context-dependent functions of individual miRNAs within complex regulatory networks. Addressing these barriers will be essential for translating miRNA-based strategies into clinical applications.

Overall, this Research Topic underscores the expanding role of miRNAs in cancer biology and highlights their potential to drive next-generation diagnostic and therapeutic innovations in precision oncology.
